# Nonischemic Central Retinal Vein Occlusion in an Adolescent Patient with Ulcerative Colitis

**DOI:** 10.1155/2011/963583

**Published:** 2012-01-29

**Authors:** Deepak Vayalambrone, Tsveta Ivanova, Aseema Misra

**Affiliations:** ^1^Department of Ophthalmology, The Ipswich Hospital NHS Trust, Heath Road, Ipswich IP4 5PD, UK; ^2^Department of Ophthalmology, Norfolk and Norwich University Hospital NHS Foundation Trust, Colney Lane, Norwich NR4 7UY, UK

## Abstract

Inflammatory bowel disease (IBD) can present with extraintestinal manifestations occasionally involving the eye. Retinal vein occlusions are rarely seen and have never been reported in the pediatric population though vascular thrombosis can be associated with IBD. Here, we present a case of what we believe is the youngest reported patient with nonischemic central retinal vein occlusion (CRVO).

## 1. Introduction

Ulcerative colitis (UC) is an inflammatory bowel disorder (IBD) of unknown etiology with an incidence of 10.4/100,000 [1]. Ocular manifestations are known to be associated with IBD [2].

## 2. Case Report

A 16-year-old female patient presented to the emergency eye clinic with a 5-day history of blurred vision in her right eye. Her past ophthalmic history was unremarkable. Her past medical history was significant for a diagnosis of UC, 3 months previously, treated initially with oral prednisolone, at a dose of 60 mg tapered down to 10 mg when she was seen in the eye clinic. She was also on Azathioprine 100 mg daily. She denied being on the oral contraceptive pill.

**Figure 1:**
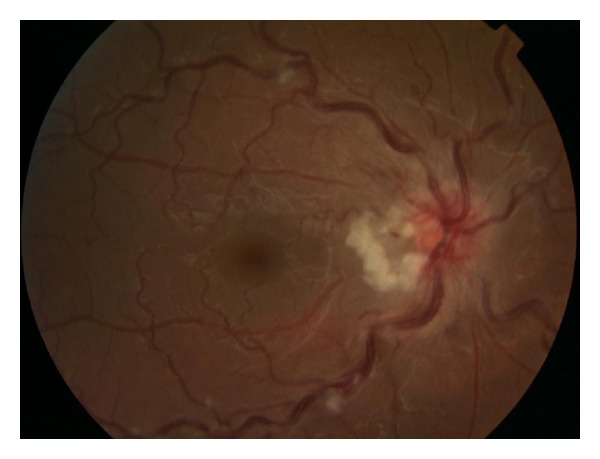
This is a picture of the fundus of the right eye showing dilatation of the veins, cotton wool spots (along the vessels), and exudates (adjacent to the optic disc).

The visual acuity was 6/5 in both eyes on checking with a Snellen's chart. There was no relative afferent pupillary defect, and colour vision testing using the Ishihara charts showed no abnormality, showing a clinically normal optic nerve function. Anterior segment examination revealed no abnormality, and the intraocular pressure was normal in both eyes. Fundus examination revealed dilated retinal veins, a few cotton wool spots, few deep retinal hemorrhages, peripapillary exudates, and mild oedema of the right optic disc (see Figure 1). Fundus examination of the left eye showed no abnormality. A diagnosis of a nonischemic central retinal vein occlusion was made. She had comprehensive range of blood tests including a full blood count, protein electrophoresis, lupus anticoagulant, autoantibodies, and thrombophilia screen all of which showed no abnormality. MRI scanning of the orbits and visual pathway showed no focal abnormality.

On followup at 3 months, she reported an improvement in the subjective visual symptoms. The visual acuity was 6/4 in both eyes, and there was a marked reduction in the exudates. The cotton wool spots had disappeared. At her final followup 15 months after the initial presentation, all retinal signs had reversed and the visual acuity was 6/4 in both eyes.

## 3. Discussion

Patients with IBD are known to develop ocular manifestations such as episcleritis, scleritis, neuroretinitis, central serous retinopathy, and orbital pseudotumours [2]. Vascular occlusions have also been reported, and IBD is thought to be associated with an increased risk of venous thrombosis [3]. We believe this to be the youngest patient with a nonischemic central retinal vein occlusion. CRVO has, however, been reported in an otherwise healthy child [4]. Subclinical retinal vasculitis may be present in patients with IBD [5]. The risk of retinal venous occlusions may relate to the activity of the underlying systemic condition, and it is important for treating physicians to be aware of this complication at least during the active phase of IBD in young patients. The central Retinal vein occlusion study reported that 34% of patients with good perfusion (nonischemic) converted to ischemic within 3 years [6]. This conversion was most rapid in the first 4 months. The results of this and other studies need to be applied with caution in the pediatric age group as they were mainly conducted on an older population. The risk of ischemic CRVO in the pediatric population is unknown but is likely to be very low as a literature search revealed no reported cases.
